# Placenta percreta - a management dilemma: an institutional experience and review of the literature

**DOI:** 10.4274/jtgga.galenos.2020.2020.0106

**Published:** 2020-12-04

**Authors:** Kavita Khoiwal, Amrita Gaurav, Dhriti Kapur, Om Kumari, Pankaj Sharma, Rekha Bhandari, Jaya Chaturvedi

**Affiliations:** 1 Department of Obstetrics and Gynecology, All India Institute of Medical Sciences, Rishikesh, India; 2 Department of Radiology, All India Institute of Medical Sciences, Rishikesh, India; 3 Department of Pathology, All India Institute of Medical Sciences, Rishikesh, India

**Keywords:** Placenta percreta, uterine artery embolization, elective delayed hysterectomy, immediate cesarean hysterectomy

## Abstract

**Objective:**

Placenta percreta is an extremely high-risk obstetric condition often associated with significant maternal morbidity and mortality. To date, there is no consensus on its management. This article aimed to identify an optimum management option to improve maternal outcomes in patients with placenta percreta.

**Material and Methods:**

This was an observational study conducted at a tertiary care institute from October 2019 to June 2020. A well-defined plan of preoperative, bilateral, uterine artery catheter placement, cesarean delivery (CD) of the baby followed by uterine artery embolization (UAE), and elective delayed hysterectomy after 2-4 weeks, was made by a multidisciplinary team. Demographic variables such as age, parity, period of gestation, presenting complaints, imaging findings, mode of management, intraoperative findings, blood loss, the requirement for blood and blood products, and complications were noted.

**Results:**

We encountered seven cases of placenta percreta over a period of nine months. UAE was performed in 6/7 patients. UAE was not performed in one patient as she presented to the emergency department in shock. Elective delayed hysterectomy was performed after 2-4 weeks in three patients, three patients required emergency hysterectomy (two during CD and one on the seventh postoperative day) and one patient was managed conservatively by leaving the placenta in situ after CD and UAE. Patients who underwent UAE had notably less intraoperative blood loss and requirement of blood and blood products than the patient who could not receive UAE. During cesarean hysterectomy, blood loss was 1,700 mL in embolized (case 4) vs 3,000 mL in unembolized patient (case 7). In embolized patients, the median blood loss during CD (case 1,2,3,5,6) was 200 mL (interquartile range: 165-200 mL) and during delayed elective hysterectomy (case 1,3,5) was 150 mL (range: 125-225 mL). Blood loss in case 2 was 1,000 mL during emergency hysterectomy on the 7^th^ day of CD and UAE. The blood loss was appreciably higher in patients who underwent immediate cesarean hysterectomy rather than elective delayed hysterectomy.

**Conclusion:**

Placenta percreta, if not managed in a preplanned manner, may lead to disastrous maternal outcomes. Prophylactic devascularization during CD and leaving the placenta in situ followed by elective delayed hysterectomy, might be a reasonable management option in most severe cases of placenta percreta.

## Introduction

Placenta percreta is one of the most dreaded obstetric complications. The overall incidence of placenta percreta is low at 5% of all placenta accreta spectrum (PAS) cases, but the incidence is currently rising owing to an increased rate of cesarean deliveries (CD). The reported incidence of PAS is 1 in 300 (1) and the risk of bladder invasion is much lower (1 in 10,000 pregnancies) (2). PAS is associated with significant maternal morbidity (24-67%) including intractable hem-orrhage (2-4 liters), bladder injury, a requirement for massive blood transfusion, disseminated intravascular coagulopathy (DIC), thromboembolism, systemic infection, sometimes repeat surgeries (3) and mortality (7%) (4). The severity of complications increases with the severity of placental invasion. Therefore, pla-centa percreta is the most dangerous manifestation of PAS.

To date, there is no consensus on the management of these cases. However, cesarean hysterectomy is the widely accepted management for PAS (5). In contrast, a conservative management option includes leaving the placenta in situ for spontaneous resorption, but it is associated with increased morbidity, high risk of infection, hemorrhage, and a requirement for emergency hysterectomy in 58% of cases within nine months of cesarean section (6). Moreover, in cases of placenta percreta, owing to the high morbidity and mortality rate, it seems reasonable to utilize alternative options such as delayed hysterectomy with or without prophylactic devascularization (7). The debate continues between a conservative or surgical approach, and immediate cesarean hysterectomy or elective delayed hysterectomy.

We report a series of seven cases of placenta percreta managed successfully at our institute. The aim was to identify an optimum management option to improve maternal outcomes in patients with placenta percreta based on the available literature.

## Material and Methods

This was an observational study conducted at the department of obstetrics and gynaecology of a tertiary care institute. Being a referral center and despite the low prevalence, seven cases of placenta percreta were encountered over a period of nine months, from October 2019 to June 2020. All cases in which the placenta was found to be invading the entire uterine wall, penetrating the uterine serosa, and encroaching adjacent organs, such as the bladder or parametrium were designated placenta percreta. The initial method of diagnosis of placenta percreta was ultrasound (USG), subsequently confirmed with magnetic resonance imaging (MRI).

**Figure 1:**
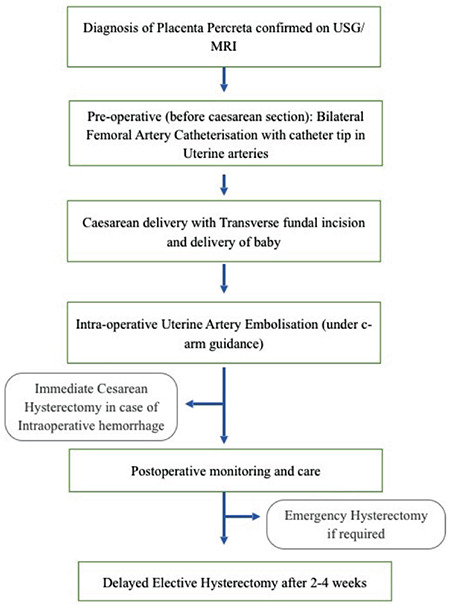
Algorithm for management of placenta percreta at our centre USG: Ultrasound, MRI: Magnetic resonance imaging

On referral of the first case, a multidisciplinary team including a senior obstetrician, interventional radiologist, urologist, transfusion medicine, neonatologist, critical care, and anesthetist was formed to avoid or minimize intraoperative hemorrhage and postoperative complications. After thorough discussion of the pros and cons of the conservative and surgical approach, a well-defined plan of preoperative bilateral uterine artery catheter placement, CD of the baby followed by uterine artery embolization (UAE) and elective delayed hysterectomy after 2-4 weeks (Figure 1) was formed. A transverse incision on the uterine fundus was planned to deliver the baby during cesarean section, as lower segment transverse or vertical incision can directly transect the placenta (8). Similarly, a vertical incision over the upper uterine segment may extend to the placental margins and lead to catastrophic hemorrhage, resulting in increased maternal-fetal morbidity and mortality.

A written and informed consent was taken from all patients. The study was reviewed and approved by the Institutional Ethical Committee (approval number: AIIMS/IEC/20/341) of All India Institute of Medical Sciences Rishikesh.

Antenatal booking status, referral, demographic variables such as age, parity, period of gestation, presenting complaints, imaging findings, mode of management, intraoperative findings, blood loss, a requirement of blood and blood products, and complications were noted.

### Statistical analysis

Baseline demographic characteristics, intraoperative, and postoperative outcome variables of all cases of placenta percreta were noted in a tabular form. Descriptive statistics were used to calculate simple frequency, percentage, and proportion. Intraoperative blood loss was calculated as median with interquartile range.

## Results

**Figure 2:**
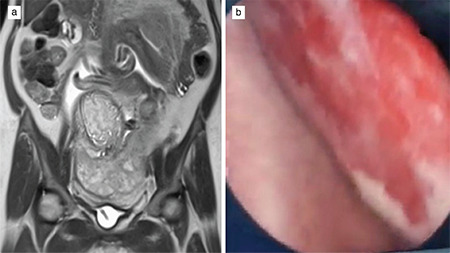
(a) T2 weighted magnetic resonance imaging (coronal section) showing placenta invading through the lower uterine segment reaching up to bladder serosa; (b) Cystoscopy showing placental bulge with intact bladder mucosa

Table 1 shows baseline demographic characteristics, imaging, and intra-operative and post-operative variables. All patients were young, with age ranging from 29 to 34 years. Most of the patients (5/7) were unbooked and either referred or presented to the emergency department. Planned elective CDs were performed at 34-36 weeks of gestation, with the exceptions of case 1 and case 7 when CD was performed at approximately 37 weeks, as they presented in late gestation (Table 1). Figure 2 shows MRI and cystoscopic image of case 1.

**Figure 3:**
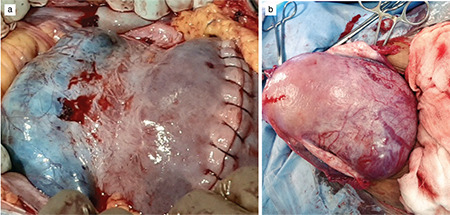
Intraoperative image of showing placenta percreta with an anterior bulge in case 1 (a) and a posterior bulge in case 2 (b)

UAE was performed in 6/7 (85.7%) patients as per our protocol. On the day of CD, bilateral femoral arteries were catheterized, with catheter tips placed bilaterally on uterine arteries under USG guidance. The patient was then shifted to the operating theatre for CD. General anesthesia was administered, and the abdomen opened in layers under asepsis. Intraoperative findings of case 1 and case 2 are shown in Figure 3. A transverse fundal incision was made to deliver the baby, cord clamped, and cut. No oxytocics were given. Signs of spontaneous placental separation were awaited. In the absence of such signs the placenta was left in situ. Then the interventional radiologist, in the operating theatre itself, confirmed the position of catheter tips on bilateral uterine arteries under C-arm guidance with subsequent embolization with gel foam until stasis was achieved. Case 4 required immediate cesarean hysterectomy because of massive hemorrhage from placental sinuses. In all other cases, after ensuring that there was no bleeding from the placental site and vagina, the uterus was closed followed by the abdomen. All surgeries were performed by the same surgeons.

Every patient was closely observed in the post-operative period. Case 2 developed significant postpartum hemorrhage (PPH) on the seventh day after surgery and was taken for emergency hysterectomy during which no intraoperative complications were faced. Elective delayed hysterectomy was successfully performed on the 14th day in cases 1 and 3 and on the 30th day of CD in case 5. Case 6 was keen for conservative management and has thus been kept on regular follow up for the last four months. Her USG suggested a regressed placenta.

UAE could not be performed in case 7 as she presented to the emergency department at midnight with antepartum hemorrhage and features of shock. Therefore, she was immediately taken for cesarean hysterectomy. Intraoperative blood loss was significant (approximately 3,000 mL) and required more than four units of packed red blood cells. In comparison, blood loss during immediate cesarean hysterectomy in an embolized patient (case 4) was 1,700 mL.

In embolized patients (cases 1,2,3,5,6), the median (interquartile range) blood loss during CD was 200 (165-200) mL and during delayed elective hysterectomy (case 1,3,5) was 150 (range: 125-225 mL). Blood loss in case 2 was 1,000 mL during emergency hysterectomy on the seventh day after CD and UAE. Intraoperative blood loss and requirement for blood and blood products are shown in Table 1.

**Figure 4:**
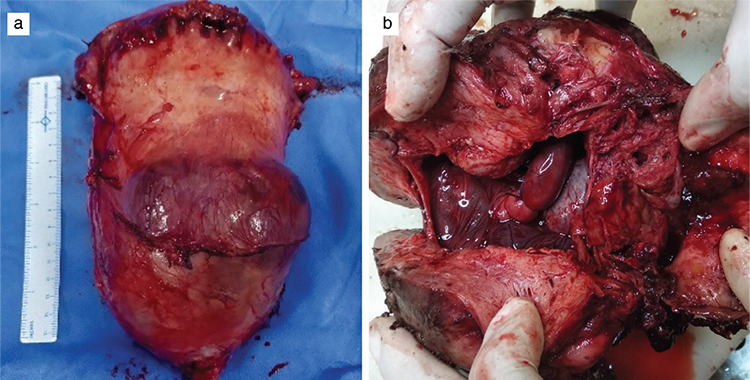
Hysterectomy specimen showing placenta percreta in case 1 (a) and 2 (b)

**Figure 5:**
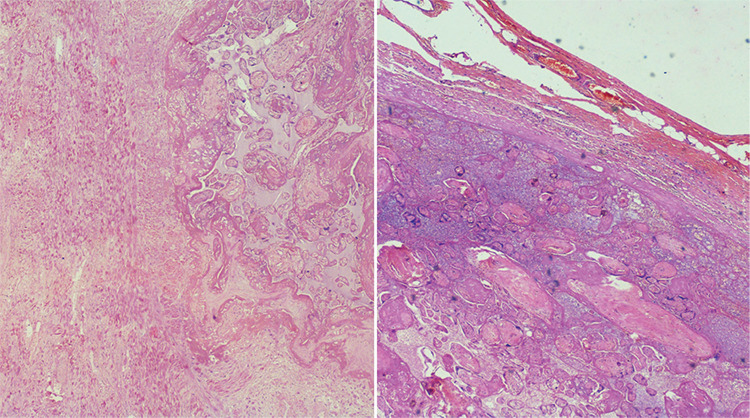
Hematoxylin and eosin (40x) stained sections of case 1, showing chorionic villi implanted into the myometrium without intervening decidua and full thickness invasion of the myometrium

Figure 4 shows uterine specimens of cases 1 and 2 at the time of delayed hysterectomy, significantly regressed in size and vascularity. All seven patients did well in their postoperative period. The final diagnosis of percreta was confirmed by histopathology. Figure 5 shows the histopathological image of case 1.

Using this planned multidisciplinary team approach, a good maternal and fetal outcome was achieved.

## Discussion

The optimal management of placenta percreta is not yet clear. A wide array of management options have been discussed in the literature, yet no preferred management modality has been identified, possibly due to the low incidence of such cases. There is an ongoing debate whether to go for cesarean hysterectomy or conservative management leaving placenta in situ with or without elective delayed hysterectomy.

Cesarean hysterectomy in cases of placenta percreta is associated with high rates of severe maternal morbidity (40-50%) and mortality (7%) (7). Similarly, high maternal morbidity (56%) during conservative management of placenta percreta has been reported by Matsuzaki et al. (9) in a systemic review. Massive hemorrhage and urinary tract injury are the most worrisome complications of cesarean hysterectomy, whereas the conservative approach is associated with late complications of leaving the placenta in situ. These late complications include secondary PPH, infection, DIC and need for an emergency hysterectomy (10). Therefore, detailed counseling of the patients, informed consent, and multidisciplinary approach for management is mandatory in these cases for an optimum outcome. We performed delayed hysterectomy wherever feasible, to avoid complications related to both approaches. Delaying hysterectomy might result in decreased uterine blood flow and regression of placenta from surrounding structures. UAE was also performed in most of the cases to minimize hemorrhage.

The exact time to perform delayed hysterectomy is debatable. The timing of elective delayed hysterectomy in our study was after 2-4 weeks following CD. In contrast, Zuckerwise et al. (7) suggested that the ideal time for hysterectomy was after 4-6 weeks subsequent to CD. However, Collins et al. (11) suggested re-evaluation of the time of delayed hysterectomy as they hypothesized that if the patient remains stable after 4-6 weeks of CD, hysterectomy may not be needed. The International Society for Abnormally Invasive Placenta also documented no added advantage of elective delayed hysterectomy, and the associated potential risks of a second elective surgery in a stable patient (12). Similarly, Matsuzaki et al. (13) reported spontaneous placental absorption in 80% of cases after 4-6 weeks of CD during a conservative approach and suggested that elective hysterectomy may not be required after 4-6 weeks of CD. However, 10% of conservatively managed cases had a massive hemorrhage, and 10% of cases developed fistula formation, arteriovenous malformation, and DIC leading to emergency hysterectomy (13). Hence, the questions “whether to plan for elective delayed hysterectomy or not” and “when” are still unanswered.

In our experience, patients who underwent planned elective surgery had lesser blood loss and morbidity than emergency surgery. Silver et al. (14) also documented better maternal outcomes with elective surgeries.

We experienced appreciably higher blood loss in patients who underwent immediate cesarean hysterectomy compared to planned delayed hysterectomy. Likewise, Zuckerwise et al. (7) reported significantly high median estimated blood loss (EBL) with immediate hysterectomy (3,000 mL) compared to a delayed hysterectomy (1,300 mL) in placenta percreta cases. The requirement of transfusion of >4 units of RBC was also significantly higher in cases of immediate cesarean hys-terectomy compared with delayed hysterectomy (p=0.016).

Ouerdiane et al. (15) in their prospective study on conservative management of placenta percreta, where no additional therapy was used, reported that almost 31% of cases were complicated by massive obstetric hemorrhage or infection and required an emergency hysterectomy. In such a scenario, the role of a conservative approach with additional procedures such as injection methotrexate and UAE to decrease vascularity may be considered as a safe management option. This further paves the way for a technically easier planned delayed hysterectomy with a reduced rate of peri- and post-operative complications. The efficacy of methotrexate is questionable in late pregnancy, as it acts only on proliferative cells and trophoblastic proliferation is absent in late pregnancy (16). Moreover, the adverse effects of the drug itself have led to discouragement in its use, whereas selective arterial embolization is reported to have a 90% success rate in cases of PAS disorders (17).

In our experience, patients who underwent UAE had a better outcome in terms of intraoperative blood loss and the requirement of blood and blood products. Although we had planned delayed hysterectomy from 2-4 weeks after CD and UAE, 2/6 patients required emergency hysterectomy. Nevertheless, the overall EBL was notably lower in them than the patient who could not receive UAE.

Wang et al. (18) also described significantly less EBL, need for blood transfusion, and length of ICU stay with UAE before hysterectomy than cesarean hysterectomy alone. On the contrary, Matsuzaki et al. (9) reported no benefit of prophylactic UAE to improve success rates, although they documented earlier placental resorption with UAE when compared to no UAE (22.4 weeks vs 35.3 weeks; p=0.014). Prophylactic devascularization, either surgically or radiologically, is expected to reduce intra-operative blood loss, secondary hemorrhage, and accelerate placental resorption (19,20).

Internal iliac artery balloon occlusion has also been found to minimize blood loss (21). Cali et al. (22) also documented a statistically significant reduction in mean EBL (933 vs 1507 cc). In contrast, Salim et al. (23) demonstrated no difference in mean EBL with internal iliac artery balloon occlusion in women undergoing CD for suspected PAS. In a review article, Kingdom et al. (24) stated that with surgical expertise, balloon placement and devascularization do not improve patient outcomes. Additionally, it is highly demanding in terms of cost and hospital resources. Furthermore, vessel occlusion itself could pose certain complications such as hampered blood supply to lower limbs and pelvis (25), buttock necrosis (26), post embolization syndrome, uterine scarring, and secondary amenorrhea (27). We did not have any UAE-related complications in our case series.

Ligation of the anterior division of internal iliac arteries during surgery is another option although this is more surgically demanding. However, Hussein et al. (28) in a randomized trial reported no additional advantage of prophylactic internal iliac artery ligation during cesarean hysterectomy. It is well known that the blood supply of the lower uterine segment, para-vesical spaces, and vagina comes from both internal iliac arteries and collaterals of external iliac arteries in percreta cases. Kingdom et al. (24) further proposed a five step approach to cesarean hysterectomy which leads to a mean EBL of less than 1.5 liters.

In the literature review, other alternatives described to minimize intra-operative blood loss were balloon placement in the infra-renal aortic and common iliac arteries. Li et al. (29) did a retrospective study to compare outcomes of all three levels of balloons (infra-renal aortic, common iliac and anterior divisions of the internal iliac arteries) in women with suspected PAS and concluded that surgery with balloons placed in the infra-renal aorta or common iliac arteries had significantly lower mean blood loss (1,000 mL) than surgery with internal iliac artery balloons (2,900 mL) and significantly lower rates of hysterectomy.

The rationale behind prophylactic devascularization (UAE) in our cases was to minimize intraoperative hemorrhage and associated morbidity whereas delayed hysterectomy was performed to avoid late complications of placenta left in situ. Our approach aimed to obviate the drawbacks of both a conservative approach and primary cesarean hysterectomy. Given the fact that most of our patients had completed their family and were not eager for uterine conservation, we could use this combined approach to ensure the least morbid management. Furthermore, one of our patients wished to conserve her uterus and fortunately had no postoperative complications after CD and UAE. Embolization techniques can also be used with a conservative approach. However, the preference of management options should be individualized until we have a consensus on the optimum approach for management. We performed delayed hysterectomy 2-4 weeks after CD, which is quite early compared to other reports. A well-designed, multicentre, randomized, controlled trial with a large sample size is needed for further validation.

## Conclusion

Placenta percreta, if not managed in a preplanned manner, may lead to disastrous maternal outcomes. Prophylactic devascularization along with cesarean delivery and leaving the placenta in situ, followed by elective delayed hysterectomy might be a reasonable management option in most severe cases of placenta percreta.

## Figures and Tables

**Table 1:**
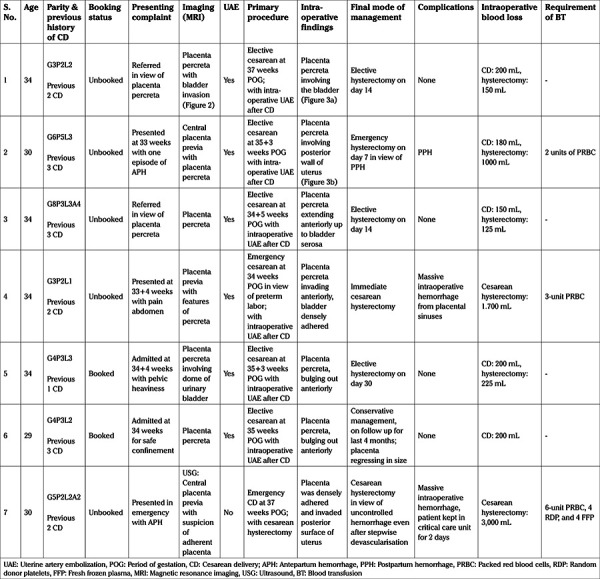
Baseline demographic characteristics, intraoperative, and postoperative outcome variables of placenta percreta cases
